# As easy as cake or a piece of pie? Processing idiom variation and the contribution of individual cognitive differences

**DOI:** 10.3758/s13421-023-01463-x

**Published:** 2023-09-19

**Authors:** Gareth Carrol, Katrien Segaert

**Affiliations:** 1 Department of English Language and Linguistics, University of Birmingham, Edgbaston, B15 2TT, Birmingham, UK; 2 School of Psychology and Centre for Human Brain Health, University of Birmingham, Birmingham, UK

**Keywords:** Idioms, Metaphor, Individual differences, Eye-tracking, Cross-modal priming

## Abstract

Language users routinely use canonical, familiar idioms in everyday communication without difficulty. However, creativity in idiom use is more widespread than sometimes assumed, and little is known about how we process creative uses of idioms, and how individual differences in cognitive skills contribute to this. We used eye-tracking while reading and cross-modal priming to investigate the processing of idioms (e.g., *play with fire*) compared with creative variants (*play with acid*) and literal controls (*play with toys*), amongst a group of 47 university-level native speakers of English. We also conducted a series of tests to measure cognitive abilities (working memory capacity, inhibitory control, and processing speed). Eye-tracking results showed that in early reading behaviour, variants were read no differently to literal phrases or idioms but showed significantly longer overall reading times, with more rereading required compared with other conditions. Idiom variables (familiarity, decomposability, literal plausibility) and individual cognitive variables had limited effects throughout, although more decomposable phrases of all kinds required less overall reading time. Cross-modal priming—which has often shown a robust idiom advantage in past studies—demonstrated no difference between conditions, but decomposability again led to faster processing. Overall, results suggest that variants were treated more like literal phrases than novel metaphors, with subsequent effort required to make sense of these in the way that was consistent with the context provided.

**Supplementary Information:**

The online version contains supplementary material available at 10.3758/s13421-023-01463-x.

Idioms, such as *a piece of cake* (meaning “really easy”), play an important role in theories of how natural language is used, processed, and learned. As (variably) noncompositional multiword expressions, they show how meaning can simultaneously be distributed over words and longer phrases; as figurative expressions, they help us understand how nonliteral meaning is dealt with; and as inherently ambiguous phrases, they show how the language processor uses multiple constraints to settle on an appropriate meaning. The current study aims to extend our understanding of how idioms are processed by focusing on two aspects that have received less attention in the existing literature: the ways in which idiomatic creativity affects processing and the contribution of individual variability in cognitive abilities to how idioms are understood.

Existing research has led to the following broad findings:

i) On the whole, idioms are recognized and understood more quickly than matched literal phrases by native speakers of a language (Gibbs, 1980; McGlone et al., 1994; Rommers et al., 2013; Swinney & Cutler, 1979). This is referred to throughout the literature as the “idiom superiority effect.”
ii) Familiarity is a key driver of idiom recognition and understanding (Carrol et al., 2018; Columbus et al., 2015; Libben & Titone, 2008; Schweigert, 1986, 1991; Schweigert & Moates, 1988; Titone & Libben, 2014). In turn, familiar idioms represent highly predictable completions, which also contributes to the fast activation of figurative meaning (Cacciari & Tabossi, 1988; Tabossi et al., 2005; Tabossi & Zardon, 1993, 1995; Titone & Connine, 1994a).
iii) Whilst initial recognition seems to be largely driven by familiarity, other factors are known to affect the selection and integration of meaning (figurative vs. literal), principally decomposability, or how much the individual words contribute to the meaning of the whole phrase (Caillies & Butcher, 2007; Titone & Connine, 1999; Titone & Libben, 2014), and literal plausibility, or whether the phrase could reasonably be interpreted literally (Cronk & Schweigert, 1992; Findlay & Carrol, 2019; Mueller & Gibbs, 1987; Titone & Libben, 2014). Individual differences are also assumed to affect this, since these (and other) factors will vary substantially across language users.

Several important caveats must be added. One is that the advantage of familiarity seems to extend to a range of “formulaic” phrases, including binomials, collocations, lexical bundles, and other frequently occurring multiword units (Arnon & Snider, 2010; Bannard & Matthews, 2008; Carrol & Conklin, 2020; Siyanova-Chanturia et al., 2011; Sonbul, 2015; Tabossi, Fanari, et al, 2009; Tremblay et al., 2011; Vilkaite, 2016). Second, processing is not “all or nothing,” and many researchers have adopted a “hybrid” position whereby idioms are subject to direct retrieval and compositional analysis simultaneously (Sprenger et al., 2006; Titone & Connine, 1999). Titone et al. (2019) demonstrated that the earliest stages of idiom processing seem to be driven chiefly by direct retrieval, whereas later stages of meaning integration demonstrate a mix of retrieval and compositional analysis, where factors such as decomposability and context interact to determine how the idiom will be understood. Lastly, despite a widespread view of idioms as “prototypically” formulaic (Siyanova-Chanturia & Martinez, 2014), corpus-based work has demonstrated that flexibility and variability are common (e.g., Fellbaum, 2019; Feyaerts, 2006; Langlotz, 2006a, 2006b; Moon, 1998; Vrbinc & Vrbinc, 2011; Wulff, 2008). How idiom variability is processed is a key question that we aim to address in the present study, with the expectation that modification (e.g., through changing component words) should require longer processing compared with canonical forms, but that individual differences in cognitive skills may affect how easily participants can retrieve the intended figurative meaning. We also aim to compare two methods that have been fruitfully applied in the literature, although rarely in tandem: eye tracking while reading and cross-modal priming.

## Idiom creativity

As mentioned above, corpus-based work has demonstrated that idiom flexibility and variability are common. Modifications to idioms often demonstrate conscious wordplay (Duffley, 2013) or explicit awareness of the citation form (Fellbaum, 2019), and a consensus is that idioms remain retrievable, provided they at least partly maintain the underlying metaphorical sense of the original (Feyaerts, 2006; Langlotz, 2006a, 2006b). These studies show that idioms can be used in a range of creative ways but tell us little about how these are processed by the intended audience. Investigations into the processing of idiomatic variants have often focused on the acceptability of syntactically and lexically modified forms. For example, Tabossi, Wolf, et al. (2009) used acceptability judgments to show that idiom syntax is determined by similar principles as literal language, with supporting context increasing the acceptability of modified forms (for similar arguments about the role of context in supporting variability, see also Fellbaum, 2019; Glucksberg, 2001; Hovhannisyan & Mkrtchyan, 2014).

Other studies have confirmed that syntactic variability is generally accepted, although the extent of this varies considerably across idioms (Tabossi et al., 2011). In online processing, some transformations (e.g., passivisation: *spill the beans* → *the beans were spilled*) do not prevent idioms from being recognized (Kyriacou et al., 2020) or the figurative meaning being activated (Mancuso et al., 2020), whereas others (e.g., adjective insertion: *spill the spicy beans*) lead to significantly longer reading times, indicating more effortful processing (Haeuser et al., 2020; Kyriacou et al., 2021). Of note here, when studies have looked at processing across different stages (e.g., comparing early vs. late eye-tracking measures), effects are much more consistently observed in later measures than during earlier stages of processing (Haeuser et al., 2020; Kyriacou et al., 2020, 2021). This in turn suggests that while initial reading of noncanonical idioms may be relatively straightforward (possibly because they were treated as literal phrases), later stages, where compositional analysis is likely to demonstrate that a literal reading is anomalous, are where slowdown is more likely to occur.

Lexical variation is generally more disruptive than syntactic variation, but the extent of this can be affected by familiarity with the underlying idiom (McGlone et al., 1994), context (Holsinger, 2013), transparency (Geeraert et al., 2017), and how close the replacement word is to the original idiom (Hamblin & Gibbs, 1999), as shown through a range of rating, reading, and visual-world eye-tracking tasks.^1^ Smolka and Eulitz (2020) used acceptability ratings to compare idioms (*reach for the stars*) and variants (*reach for the planets*). Ratings for variants were lower than for canonical idioms but higher than for unrelated control forms. They also found that modification of nouns led to lower acceptability ratings than modification of either verbs or prepositions.

A related line of research has shown that even when translated from another language, idioms remain recognizable and interpretable (Carrol et al., 2016; Carrol & Conklin, 2014, 2017; Senaldi et al., 2022; Titone et al., 2015), suggesting that a specific surface form is not the only possible route to idiom processing. Conversely, when idioms are entirely unfamiliar, processing is shown to be highly disrupted as language users must actively engage in the process of working out the intended meaning. Carrol and Littlemore (2020) showed that entirely unknown idioms came with a predictable cost during natural reading and were read significantly more slowly than literal paraphrases. Importantly, more transparent phrases had shorter reading times in a way that was not seen for known idioms or conventional metaphors (see also Libben & Titone, 2008, for evidence that decomposability has a limited effect on highly familiar idioms). The literature on metaphor processing also shows that factors such as familiarity and conventionality are primary drivers of understanding (Blasko & Briihl, 1997; Inhoff et al., 1984), while aspects relating to interpretability only play a role in less familiar phrases (Blasko & Connine, 1993).^2^

### Individual cognitive components of idiom processing

As in language processing more generally (e.g., Kidd et al., 2018), interest in the role of individual cognitive differences has provided a further set of questions to develop our understanding of how figurative language is understood. Evidence here has come from two sources: neuropsychological studies (of e.g., language impaired individuals) and studies measuring the impact of individual components of cognitive function on various language tasks.

For idioms, a series of investigations by Papagno and colleagues (Oliveri et al., 2004; Papagno et al., 2003, 2004, 2006; Papagno & Caporali, 2007; Papagno & Genoni, 2004) highlighted the importance of executive function (the suite of abilities that underpins the brain’s ability to monitor and manage cognitive processes in a flexible, adaptable way) in processing figurative phrases amongst patients with Alzheimer’s disease and aphasia. Executive function may play a vital role here since successful idiom interpretation requires the language user to consider and disambiguate competing meanings, maintain relevant information in working memory, inhibit irrelevant meanings, and so on. In general, impairment led to more literal interpretations of idioms, and the authors suggested that this was due in part to an impaired ability to ignore the (less relevant) literal meanings in the contexts provided. For example, patients with mild Alzheimer’s disease performed poorly on idiom comprehension during a sentence-to-picture matching task when a figurative versus literal interpretation of an idiom was offered, but performance improved significantly when the same task offered a figurative versus unrelated picture choice, and when asked to provide verbal explanations of idiom meanings (Papagno et al., 2003)

Amongst nonclinical participants, studies have largely focused on the role of working memory, and how this affects the ability to maintain and select between competing figurative and literal meanings. In metaphor studies, high working memory has been consistently linked to faster and more successful interpretation (Blasko, 1999; Carriedo et al., 2016; Chiappe & Chiappe, 2007; Kazmerski et al., 2003; Olkoniemi et al., 2016; Pierce et al., 2010). Columbus et al. (2015) also looked at the role of executive control, focusing on individual differences amongst healthy participants and their effect on processing of metaphors and idioms. Their results suggest a pattern whereby higher executive control led to longer initial reading times for metaphors, indicating a tendency for readers to take information into account and commit to a particular interpretation of a potentially metaphorical verb as they were reading it. In comparison, participants with lower levels of executive control spent less time overall on metaphors (compared with literal controls) but were more likely to regress to a preceding context to help with interpretation. Idiom processing showed no such effects of executive control, supporting the view that they were retrieved wholistically (with no cognitive control required) rather than analyzed/constructed online.

Cacciari et al. (2018), in an exploratory study, found that online idiom comprehension (response times in a cross-modal lexical decision task) was positively affected by higher scores for working memory, inhibition control and crystalized verbal intelligence. They argue that successful processing of idioms requires activation of multiple sources of information (literal meanings of words as well as the overall idiomatic meaning) as well as an ability to suppress the literal meaning once this is shown to be irrelevant. The authors also suggest that, as with language acquisition and processing more generally, a large vocabulary is required for this to be completed efficiently. To summarize the findings so far, links between working memory and metaphor processing in healthy adult language users have been demonstrated in several previous studies, with effects in both early and later measures of processing, while evidence for links between working memory or executive/inhibitory control and idiom processing are still tentative. An open question is how modified idioms of the type investigated in the studies discussed earlier in this section might therefore be affected by these variables, since they are neither entirely novel (like metaphors) nor entirely familiar (like idioms).

In the current study we focus on idiom variation, and the contribution of individual differences in cognitive function to how this is processed. Only one previous study has investigated this issue, albeit indirectly. Haeuser et al. (2020), as discussed in the previous section, compared processing of canonical versus modified (inserted adjective) idioms. They also compared idiom processing for younger and older adults, to compare contributions of fluid cognitive functions (working memory, processing speed, inhibition control) with crystallized cognitive abilities such as better world and lexical knowledge (including a more entrenched knowledge of idioms).^3^ They found evidence that older adults accessed canonical idioms more quickly than younger participants (relative to literal controls), and that subsequent literal biasing context caused more difficulty for older readers, which they interpreted as indicating difficulty in suppressing a figurative meaning when this was necessary. Modified forms of idioms caused similar problems for older and younger participants, with both groups showing a bias toward a literal reading. As above, effects of modification were most apparent in later measures of processing, which is where the various idiom and cognitive factors discussed so far may have the biggest role to play (as opposed to earlier, “retrieval-based” stages—Titone et al., 2019).

### Summary and hypotheses

The literature discussed above broadly reveals a picture where creative variation does not necessarily block access to idiom meanings but does lead to longer processing times, assumed to represent the more effortful process of reconfiguring the phrase to reach the intended meaning. The aim of the current study is to investigate the online processing of idioms and creative modifications. We predict that modifying idioms by changing the final noun will lead to longer processing times, but that individual differences in cognitive variables such as working memory and inhibitory control may affect how easily modifications are processed. Similarly, individual properties of idioms may attenuate this process. More decomposable idioms may be more amenable to modification, since the underlying figurative meaning should be less disrupted than for less decomposable phrases. If variants are initially treated as literal phrases, then these effects (slower processing for modified forms but also the influence of both cognitive and idiom variables) should be most apparent during later stages of processing, where compositional analysis and meaning integration are assumed to play a more prominent role. Conversely, if variants are immediately recognized as either modifications of known phrases or as novel metaphors in their own right, the earliest lexical recognition stages may also be impacted. Individual familiarity may also reduce disruption, if highly familiar idioms can be more easily accessed and reconfigured, or, conversely, may serve to make modification more disruptive, if more familiar idioms are more firmly entrenched. Effects here may show up in both early (idiom recognition) and late (reconfiguration and analysis) stages of processing.

## Method

The study uses two of the principal behavioural methods for investigating idioms: eye tracking while reading and cross-modal priming (see Carrol, 2021, for a review of these methods in figurative language research). Eye-tracking records eye movements during natural reading, with both duration and sequence of fixations contributing to an understanding of how text is processed. Fixation measures are often divided into “early,” reflecting the initial processes of word recognition/contact with the lexicon, and “late,” reflecting the process of meaning selection/integration (Altarriba et al., 1996; Conklin et al., 2018; Godfroid, 2020; Inhoff, 1984; Paterson et al., 1999; Rayner, 2009). This provides a way to infer the ease or difficulty with which idioms are processed and has helped to show the difference between recognizing a known phrase (through early measures such as first pass reading time) and making sense of the meaning (through later measures such as total reading time and regressions back into an idiom).

Cross-modal priming (Swinney, 1979; Swinney et al., 1979) has been used to demonstrate the activation of figurative or literal meaning during the processing of idioms. Participants listen to a spoken sentence and then at a key point are presented with a visual target word and asked to make a lexical decision (Is this a real word, yes or no?) as quickly as possible. In idiom studies, targets are typically related to figurative or literal meanings of the idiom, and compared with a baseline (an unrelated word) to assess the degree and/or time course of activation. A common finding is that responses to figuratively related words are faster than to unrelated words, which is taken as evidence for the rapid activation of idiom meanings for well-known phrases. These two methods have each been fruitfully applied in the study of idioms but are rarely used together, and offer different perspectives on this issue. Whilst cross-modal priming offers a way to directly measure activation of meaning, eye tracking provides a more inferential account whereby we can assess the factors that make processing in context easier or harder. Our experimental session therefore incorporated a reading study and a cross-modal lexical decision task, to directly compare results from the two.

### Participants

Forty-seven participants took part (42 females, mean age = 19.3 years, *SD* = 1.3) and everyone completed all aspects of the study. Participants were all monolingual native speakers of English, with normal or corrected-to-normal vision and no self-reported history of speech or language disorders. Half of the participants completed the reading task first and half completed the cross-modal priming task first. In between the reading and cross-modal priming task, a series of cognitive tests was administered, and following all tasks a familiarity test was administered to assess how well each participant knew the idioms they had seen during the study. Overall, participants took around 1 hour to complete all tasks.

### Materials

Fifty-one idioms of the form “verb-X-noun” (e.g., *play with fire*) were selected from previously published studies (see the Supplementary Materials for a detailed description of item selection). We chose to investigate lexical variation as this has had the clearest impact on idiom comprehension, based on previous work. For all items we created a plausible variant by replacing the final noun in a way that maintained the metaphorical sense of the idiom. For example, in *play with fire*, we replaced *fire* with *acid*, to maintain the underlying meaning of *play with* [*dangerous thing*]. A literal control phrase for each idiom was also created in the same way, hence for *play with fire* the control was *play with toys*. Nouns were matched as closely as possible for length and individual frequency (from the British National Corpus). Ratings for literal plausibility, decomposability, semantic similarity between idiom and variant nouns, and acceptability of variants were collected for all items. Table 1 summarizes the characteristics of the stimuli, and a full list of experimental items is provided in the Appendix.

**Table 1:** Characteristics of stimuli, including frequency (on the Zipf scale of 1–7^1^) of final nouns for idioms, variants and literal phrases; decomposability ratings (1–5) for idioms (Idiom Decomp); semantic similarity scores (0–1) between idioms and variants; and substitutability ratings (1–5) for idioms and variants

	Idiom Noun freq	Variant Noun freq	Literal Noun freq	Idiom Decomp	Semantic Similarity	Substitutability
Mean (*SD*)	4.67 (0.57)	4.65 (0.53)	4.67 (0.52)	3.06 (0.77)	0.54 (0.27)	3.04 (0.69)
Range	2.83–5.63	2.57–5.51	3.20–5.58	1.64–4.62	0.13–1.00	1.4–4.5

^1^The Zipf scale (van Heuven et al., 2014) is a logarithmic scale reflecting relative frequency given the size of the corpus being used. A value of 1 represents 1 occurrence per 100 million words, 2 represents 10 occurrences per 100 million words, 3 represents 100 occurrences, and so on.

For the reading study, we created context sentences that supported either the idiom or variant meaning (same context for both) or the literal phrase. All sentences consisted of two conjoined clauses. The first clause always began with a personal pronoun and introduced the target phrase in a neutral way, followed by the target phrase (e.g., *They have both been playing with* [*fire / acid / toys*]). The second clause followed a conjunction and was consistent with the figurative (e.g., *and it’s no surprise that things have gone badly*) or literal (e.g., *and seem to have been getting on very well together*) version of the phrase. Fifty-one filler items were created, comprising literal sentences of approximately the same length and syntactic structure as the critical sentences. Items were counterbalanced over three presentation lists, with an idiom, its variant, and its literal control phrase appearing on different lists. Each list contained 17 items in each condition, and the same filler sentences were used in all three presentation lists, for a total of 102 items per list.

For the cross-modal priming, a word related to the figurative meaning of the idiom was chosen (e.g., for *play with fire* the target was “danger”), matched across all items for length and frequency. The sentences created for the reading task were all reused for the cross-modal priming task, up to the end of the key phrase (e.g., for *play with fire* the stimulus item in the idiom condition was *They have both been playing with fire*). Filler items from the reading task were adapted by cutting the sentence off after the first clause, to leave items of around the same length as the critical stimuli. Filler items were always paired with a pseudoword. We also added in an additional 17 unused idioms, to give a total of 68 fillers. The purpose of this was to ensure that some (noncritical) idioms could be followed by a pseudoword to avoid participants developing expectancy-based strategies. The critical and filler items were used to create sound files using an online voice synthesizer recorded with a female voice with a British English accent. Critical items were paired with their related word and counterbalanced over the same three presentation lists as in the reading study, meaning that 17 idioms (critical items), 17 variants, and 17 literal controls were followed by a real word, and 68 fillers (51 literal sentences and 17 idioms) were followed by a pseudoword. Pseudowords were all pseudohomophones between four and eight letters long, created using the ARC Nonword Database (Rastle et al., 2002).

Participants were randomly assigned to one of the combinations of presentation lists (AB, AC, BC) so that no one saw the same list for the two main tasks —that is, if a participant saw *play with fire* in the reading task, they would see either *play with acid* (variant) or *play with toys* (literal) in the cross-modal priming task. Whilst the use of two tasks with overlapping stimuli opens up the possibility of priming effects between the two, the use of counterbalanced presentations lists should ensure that these effects were minimized.

### Reading task

Participants were seated in front of a desk-mounted EyeLink 1000+ eye tracker (SR Research). A chin rest was used to ensure head stability during the task. Following a nine-point calibration and validation procedure, participants were told that they would be presented with a series of single sentences that they should read as naturally as possible for comprehension. Recording was monocular at 500 Hz. Each trial was preceded by a fixation point, then the sentence was displayed in the middle of a white screen (47.5cm × 27cm, 1,680 × 1,050 pixels) in Courier New font size 17 pt. Forty items (10 per condition and 10 fillers) were followed by a simple yes/no comprehension question. Following six practice items, all 102 trial sentences were presented in random order. A self-timed break was offered midway through, and recalibrations were performed after the practice items, at the mid-point, and as required during the session.

### Cross-modal priming

The cross-modal priming study was built and run in E-Prime (Version 2.0). Participants were told that they would hear a spoken sentence played through speakers and immediately afterward would see a word appear on the screen. They should indicate as quickly as possible whether what they saw was a real English word using buttons marked Y and N on a serial response button box. The target word appeared 250 ms after the offset of the spoken phrase in the centre of a white screen (47.5cm × 27cm, 1,680 × 1,050 pixels) in Courier New font, size 18 pt. Based on previous studies, it was expected that idiom meaning was likely to be available immediately at the offset of the phrase for familiar items, but that figurative meaning for variants may take some time to emerge, hence a short delay was included. Participants saw eight practice items of the same form as the experimental stimuli, with a mix of word and pseudoword targets, then all items were presented in random order.

### Cognitive tests

We chose three aspects of cognitive function, based on the literature discussed previously: inhibitory control, processing speed, and working memory. Whilst working memory and inhibitory control have been included in previous idiom studies (as outlined earlier), we included processing speed in order to obtain a general idea of how quickly participants were able to process information, and how this varied in the different conditions. The cognitive tests were administered following the first task, in order to provide a distraction before participants began the second task (and to further reduce the risk of priming effects between the two main tasks). All participants completed the cognitive tasks in the same order. First, they were asked to complete a flanker task (which measures inhibitory control), programmed and delivered in E-Prime (Version 2.0). Here, participants are shown a series of five chevrons (>) pointing left or right and must indicate which way the middle one is pointing using buttons marked Left and Right on a serial response box. Trials are either congruent (where all point the same way, e.g., > > > > >) or incongruent (where the middle chevron points in a different direction, e.g., < < > < <). Performance on the incongruent trials is taken to reflect the ability to inhibit irrelevant information (the arrows pointing in the opposite direction), and the overall score is calculated as the difference in performance between congruent and incongruent trials, where a smaller difference indicates greater inhibition ability.

Second, a processing speed test was administered, using a version of the digit symbol substitution test (DSST) taken from the Wechsler Adult Intelligence Scale, Fourth UK Edition (WAIS-IV UK: Wechsler, 2010). This task presented participants with the numbers from 0 to 9 at the top of a page, along with a key assigning a symbol to each number. The page presents 135 numbered boxes and asks participants to draw the correct symbol for each, using the key provided. Following some demonstration items, participants have 2 minutes to complete as much of the test as possible, and the number of items correctly completed is taken as a measure of processing speed (max = 135).

Finally, participants were asked to complete two tests of working memory: a backward digit span task, where participants are required to repeat a sequence of numbers back in reverse order (e.g., if given “1–2”, a participant would answer “2–1”); and a subtract two task, where participants are given a sequence of numbers and must subtract two from each digit in the sequence (e.g., if given “5–3”, a participant would answer “3–1”). In both tasks, the size of the sequence increases until participants can no longer reliably provide the required numbers, and the overall working memory span is calculated as the maximum sequence size for which they could complete at least three examples out of a block of five. Overall working memory was calculated as the average of the two scores for each participant.

### Familiarity test

Finally, participants completed a familiarity test to indicate how well they knew the items used in the main studies. All 51 idioms were presented in their citation forms and participants were asked to indicate on a 6-point scale whether they knew the idiom (where 0 = *I have never heard this idiom before*) and, if so, how familiar they were with it (where 1 = *I have heard it but I’m not sure what it means*, and 5 = *I know this idiom very well and am entirely sure of what it means*).

## Results

Eye-tracking data was unusable due to technical issues or consistently poor calibration for six participants, leaving a total of 41 subjects for the reading task and 47 subjects for the cross-modal priming task. Cognitive test and familiarity data was available for all subjects and is summarized in Table 2. No significant correlations existed amongst the four variables.

**Table 2:** Inhibition score (difference between congruent and incongruent flanker trials, in ms, with scores closer to 0 representing better inhibition), working memory (average of the two tests showing the longest sequence successfully repeated), processing speed (coding score out of 135), and familiarity score (average out of 5, including a score of 0 for unknown phrases) for all participants

	Inhibitory control (flanker score)	Working memory (average)	Processing speed (coding score)	Familiarity
Mean (*SD*)	103 (42)	3.6 (0.8)	83 (12)	3.9 (0.6)
Range	35–248	2.3–6.0	56–110	2.4–4.9

For inhibitory control, all incorrect responses and RTs of 0 ms were removed; no response above 2,000 ms was recorded, and this was taken to be an acceptable upper limit.

We analyzed the eye-tracking and cross-modal priming data separately, using linear mixed-effects models in R (Version 4.2.1) and RStudio (Version 2002.07.0) using the packages lme4 (Version 1.1-30) and lmerTest (Version 3.1-3). We applied the following analyses for each study:

1. Initial analysis to compare patterns between conditions. Condition was treatment coded with literal phrases as the baseline to compare deviation from this for idioms and variants. We then used the emmeans package (with Bonferroni adjustment for multiple comparisons) to compare idiom and variant conditions directly.
2. Inclusion of the idiom variables familiarity, decomposability, and literal plausibility.
3. Inclusion of the cognitive variables inhibitory control, processing speed, and working memory.

In all analyses, the data were collapsed across lists, and all continuous variables were centred and scaled prior to inclusion. We retained trial order and length and frequency (of final nouns for the reading data and of target words for the cross-modal priming data) as covariates in all models.

### Eye-tracking analysis

Overall accuracy on comprehension questions was 90%, indicating that participants had paid attention throughout. Eye-tracking data were checked visually for accuracy, and trials where track loss was evident or where the whole of the critical phrase received no fixations were removed. This led to the loss of 3.4% of the data, leaving 2,019 trials for analysis. Since all reading took place on a single line, data were also vertically aligned to correct any minor deviations in terms of the *y*-position. The data were then cleaned according to the four-stage process in the Eyelink DataViewer software (SR Research), which removes extreme values (fixations below 80 ms and over 800 ms). Any item where a participant had indicated that they did not know the underlying idiom (score of 0 on the familiarity task) was also removed. This led to the removal of a further 159 trials (7.9% of the usable data), leaving 1,860 trials for analysis.

Table 3 presents reading data for literal control phrases, idioms, and variants. The data summarises a range of duration (first-pass reading time, total reading time, regression path duration), skipping (likelihood of skipping the final noun), and regression measures (percentage of trials with a regression into or out of a given region) for three areas of interest: the whole of the critical phrase, the final noun of each phrase, and the post-phrase region (everything following the critical phrase, to the end of the sentence).

**Table 3:** Summary of reading data for literal control phrases, idioms, and variants

	Phrase			Final noun				Post-phrase	
	Literal	Idiom	Variant	Literal	Idiom	Variant	Literal	Idiom	Variant
Skip rate %	–	–	–	0.22 (0.42) [0.19, 0.25]	0.22 (0.41) [0.19, 0.25]	0.18 (0.39) [0.15, 0.22]	–	–	–
First-pass RT (gaze duration)	458 (219) [442, 476]	431 (189) [416, 447]	463 (231) [ 446, 482]	190 (113) [181, 198]	183 (113) [174, 192]	210 (114) [201, 219]	1364 (643) [1310, 1420]	1403 (646) [1350, 1460]	1362 (685) [1310, 1420]
Total RT	596 (290) [574, 619]	550 (252) [531, 571]	670 (333) [646, 697]	220 (150) [208, 232]	204 (140) [193, 215]	267 (192) [252, 283]	1598 (690) [1550, 1660]	1564 (684) [1510, 1620]	1650 (670) [1600, 1710]
Regression path (go-past time)	595 (362) [568, 624]	566 (354) [541, 596]	625 (359) [600, 656]	266 (282) [246, 291]	236 (211) [221, 254]	291 (255) [272, 312]	1758 (690) [1690, 1830]	1692 (684) [1630, 1760]	1863 (670) [1800, 1940]
Regressions out %	0.18 (0.38) [0.15, 0.21]	0.17 (0.38) [0.14, 0.20]	0.21 (0.41) [0.18, 0.25]	0.17 (0.37) [0.14, 0.20]	0.15 (0.36) [0.12, 0.18]	0.20 (0.40) [0.17, 0.23]	0.31 (0.46) [0.28, 0.35]	0.27 (0.44) [0.24, 0.30]	0.39 (0.49) [0.35, 0.43]
Regressions in %	0.18 (0.39) [0.15, 0.21]	0.14 (0.35) [0.11, 0.17]	0.23 (0.42) [0.20, 0.26]	–	–	–	–	–	–

Duration measures are in milliseconds and figures for final nouns include zeroes for items skipped during first-pass reading; final word skip and regressions out/in reflect the proportion of trials where these occurred. Figures are mean values, with standard deviations in round brackets and 95% CIs in square backets. Skipping rate = % of words not fixated during first-pass reading; First pass RT = total time spent reading a word or phrase before exiting to the right or left; Total RT = total time spent reading a word or phrase during the trial (first-pass RT + any rereading); Regression path = total time spent reading a word or phrase and any preceding material before exiting to the right; Regressions in/out = proportion of trials where a regression was made into or out of a specified area.

We constructed initial models for all of the variables listed in Table 3. Duration measures were log transformed prior to analysis, and for any models involving binary variables (final word skip and regressions in), a generalized linear model with binomial distribution was used. All standard models included the fixed effect of condition and the covariates described above, and included random intercepts for subject and item, and by-subject and by-item random slopes for the effect of condition.^4^ We also checked substitutability scores (how acceptable variants were in conveying the figurative meaning of an idiom), but they made no improvement to any model and hence were left out. For final nouns, models for duration measures only considered items that were not skipped (hence would have durations of 0). In all models we applied a Bonferroni correction since multiple correlated measures were being tested, following the recommendations of von der Malsburg and Angele (2017). In practice, this meant adopting a new threshold for significance obtained by dividing the usual value of *p* = .05 by the number of measures being tested. For whole phrases (five measures) this gave an adjusted threshold of *p* = .01; for final nouns (five measures) *p* = .01; and for the post-phrase region (four measures) *p* = .125. For simplicity, this means that in the results that follow a *p*-value of < .01 is taken to represent a true difference.

For the whole phrase, idioms were read no more quickly than literal control phrases in first pass reading time (β = −0.06, *t* = −2.04, *p* = .045), total reading time (β = −0.07, *t* = −2.54, *p* = .014) or regression path duration (β = −0.05, *t* = −1.60, *p* = .120), and there were no differences between idioms and literal controls for regressions out of the phrase (β = −0.02, *z* = -0.11, *p* = .911) or regressions back in from later in the sentence (β = −0.26, *z* = −1.30, *p* = .195). Compared with literal control phrases, variants were read no more slowly during first pass reading (β = −0.01, *t* = −0.32, *p* = .749) and showed no difference in regression path durations (β = 0.06, *t* = 1.93, *p* = .063), but had significantly longer total reading times (β = 0.11, *t* = 3.00, *p* = .004). Regressions out of or into the phrase were no less likely in variants than literal controls (regressions out: β = 0.36, *z* = 1.96, *p* = .050; regressions in: β = 0.34, *z* = 1.47, *p* = .141). Pairwise comparison of idioms and variant phrases showed no difference in first-pass reading (β = −0.05, *t* = −1.48, *p* = .450), regressions out (β = −0.39, *z* = −1.99, *p* = .137), or regressions in (β = −0.60, *z* = −2.52, *p* = .034), but total reading time (β = −0.18, *t* = −5.34, *p* < .001) and regression path duration (β = −0.11, *t* = −3.60, *p* = .003) were both shorter for idioms.

For final nouns, no measures showed a significant difference for idioms compared with literal control phrases: skipping rate (β = 0.14, *z* = 0.74, *p* = .462); first pass reading times (β = −0.01, *t* = −0.32, *p* = .750); total reading times (β = −0.04, *t* = −1.55, *p* = .125); regression path durations (β = −0.07, *t* = −1.65, *p* = .108); and regressions out (β = −0.19, *z* = −0.90, *p* = .368) Likelihood of skipping the final word was no different in variants than literal controls (β = −0.26, *z* = −1.04, *p* = .301), nor was the likelihood of a regression from the final noun (β = 0.23, *z* = 1.15, *p* = .251). Neither first pass reading times for final nouns (β = 0.05, *t* = 1.83, *p* = .074) nor regression path durations (β = 0.05, *t* = 1.33, *p* = .192) were different for variants, but total reading times were significantly longer (β = 0.11, *t* = 2.67, *p* = .010). Comparison of idiom and variant conditions showed that neither skipping (β = 0.40, *z* = 1.86, *p* = .189) nor regressions out (β = −0.43, *z* = −2.12, *p* = .102) were more likely in idioms. First pass reading time was also no different (β = −0.06, *t* = 2.49, *p* = .055), but both total reading time (β = −0.15, *t* = −4.27, *p* < .001) and regression path duration (β = −0.12, *t* = −3.51, *p* = .005) were significantly shorter for idioms.

In the post-phrase region, idiom sentences were no different in first pass reading (β = 0.03, *t* = 0.89, *p* = .384), total reading time (β = −0.03, *t* = −0.97, *p* = .330) or regression path duration (β = −0.04, *t* = −1.22, *p* = .230) compared with literal controls, and regressions out of the post-phrase region were no more likely in idioms (β = −0.17, *z* = −0.97, *p* = .332). Variant sentences were no different to literal controls in first pass reading (β = −0.05, *t* = −1.05, *p* = .301), total reading times (β = 0.03, *t* = 1.12, *p* = .269) or regression path durations (β = 0.06, *t* = 1.96, *p* = .055), but regressions out of the post-phrase region were significantly more likely in variants than literal phrases (β = 0.49, *z* = 2.64, *p* = .008). Comparing idioms and variants directly, there was no difference in first pass reading time (β = 0.08, *t* = 1.69, *p* = .298) or total reading time (β = −0.06, *t* = −2.99, *p* = .017), but regression path durations were significantly shorter in idioms (β = −0.10, *t* = −3.85, *p* = .002) and regressions out of the region more likely for the variants (β = −0.67, *z* = −3.83, *p* < .001).

**Fig. 1:**
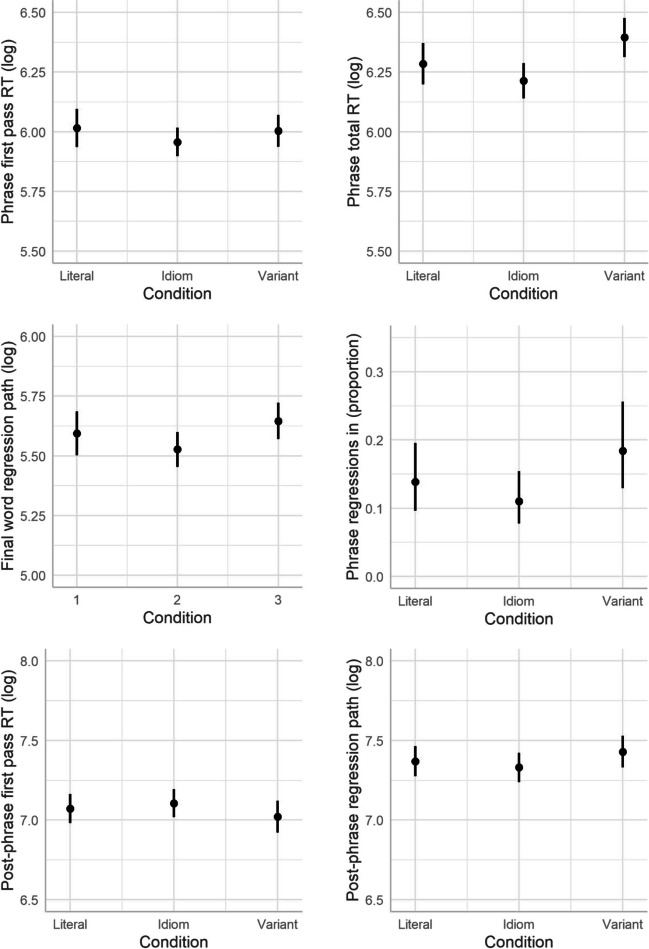
Predicted effects for phrase, final noun and post-phrase reading for literal, idiom, and variant phrases. Error bars show 95% confidence intervals

The results overall show limited differences between conditions in early measures. Crucially, this means that variant phrases were not read for longer than idioms or literal phrases on first reading. This suggest that readers may simply have treated them as literal (albeit sometimes slightly unusual) phrases on first encounter. In later measures differences emerged whereby readers spent longer on variant phrases, compared with both idioms and literal controls. Specifically, participants spent longer reading and rereading the whole phrase and final noun of critical phrases in variants than in other conditions.^5^ Readers also spent longer overall reading the rest of the sentence in variant conditions, compared with idioms, and regressions out of the post-phrase region were significantly more likely in variants than in either other condition. The main patterns are shown in Fig. 1 (predicted effects extracted from models described above).

### Idiom variables

We chose three measures for follow-up analysis, to reflect both early and late reading patterns: whole phrase first pass and total reading time, and post-phrase regression path duration. We added each of familiarity, decomposability and literal plausibility (all centred to help with model fitting) to each model, first as a fixed effect then as an interaction with condition, and used log-likelihood tests to assess any improvement. As before, since we are comparing multiple measures, we applied a Bonferroni correction to reduce the likelihood of Type 1 errors, hence used an adjusted *p* value of .05/3 = .017 (rounded up) as the threshold for significance. For brevity, we report only measures where a significant improvement was observed.

**Fig. 2:**
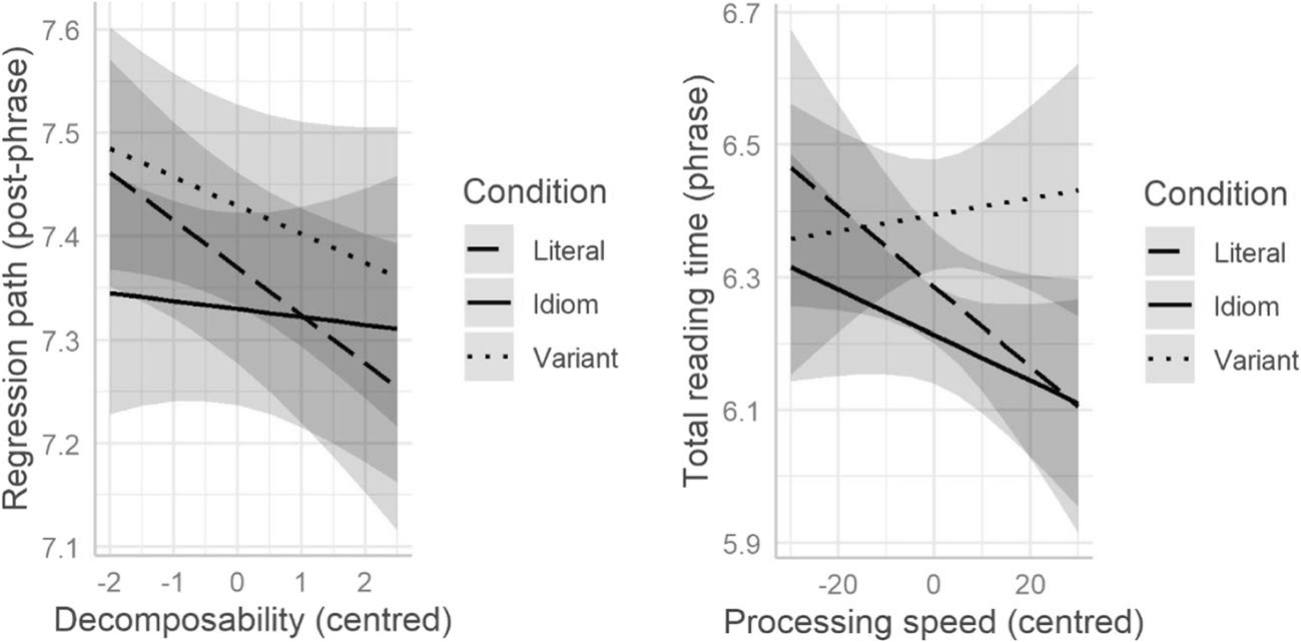
Left panel: Effects of decomposability on post-phrase regression path duration. Right panel: Effects of processing speed on phrase total reading times

Neither familiarity nor literal plausibility made any improvement to any of the models, either as a fixed effect or interaction with condition. Decomposability improved the model as a fixed effect for post-phrase regression path duration, χ^2^(1) = 5.76, *p* = .016, with no further improvement as an interaction with condition. Figure 2 (left panel) shows that higher decomposability ratings led to less time overall spent reading the post-phrase context portion of the sentence (including time spent rereading earlier parts of the sentence) across all conditions (β = −0.03 *t* = −2.52, *p* = .016).

### Cognitive variables

We next assessed the contribution of cognitive variables (centred). In each case, we added each variable to the original (condition only) model, first as a fixed effect then as an interaction with condition, and used log-likelihood tests to assess any improvement. As above, a corrected threshold of *p* = .017 was used to account for the use of multiple measures.

Neither inhibitory control nor working memory made any improvement to any model. Processing speed significantly improved the model for phrase total reading time as an interaction with condition, χ^2^(2) = 12.02, *p* = .002. Figure 2 (right panel) suggests that processing speed may have operated differently for variants, compared with idioms and literal controls.

### Three-way interactions for idiom and cognitive variables

Finally, for models where idiom variables were significant, we added in cognitive variables to check for any three-way interactions. The model for post-phrase regression path duration (including a fixed effect of decomposability) was not improved by the addition of any of the cognitive variables (as fixed effects or as interactions with condition, or as part of a three-way interactions with condition and decomposability).

### Cross-modal priming analysis

Table 4 summarizes cross-modal priming data for accuracy and response times (RTs) to the figuratively related words. Accuracy was at ceiling across all three conditions, so we focused analysis on RTs. We first removed any incorrect responses, then removed any RTs longer than 800 ms, leading to the removal of 8% of the data and leaving 2,210 trials for analysis.

**Table 4:** Summary of cross-modal priming data for idioms, variants and literal control phrases. Response times (RTs) are in milliseconds and reflect correct answers and answers <800 ms only

Cross-modal priming	Phrase		
	Literal	Idiom	Variant
Accuracy (%)	0.99 (0.12) [0.97, 0.99]	0.99 (0.06) [0.98, 0.99]	0.99 (0.10) [0.98, 0.99]
RT	532.8 (88.8) [526, 540]	525.8 (87.0) [519, 533]	532.9 (89.4) [526, 540]

Figures are mean values, with standard deviation in round brackets and 95% CIs in square brackets.

RTs were log-transformed to reduce skewing. We constructed a linear mixed-effects model with the fixed effect of condition and covariates of length and frequency (of the target word), degree of relatedness between the idiom and its target word and trial order. We included random intercepts for subject and item, by-subject random slopes for the effects of condition and relatedness, and by-item random slopes for the effect of condition. No differences were observed between literal controls and idioms (β = 0.01, *t* = −1.57, *p* = .127) or literal controls and variants (β = 0.00, *t* = 0.21, *p* = .838). Neither substitutability rating nor semantic similarity between idioms and variants made a significant improvement to the model so were excluded from all further analysis.

### Idiom variables

We next considered the same idiom variables as in the eye-tracking analysis. Neither familiarity nor literal plausibility made any improvement, but decomposability improved the model as a fixed effect, χ^2^(1) = 4.88, *p* = .027. Here, higher decomposability sped up response times for all phrase types (β = −0.01, *t* = −2.51, *p* = .013).

### Cognitive variables

We next added the cognitive variables one by one. Neither inhibitory control nor working memory made any improvements. Processing speed made an improvement as a fixed effect, χ^2^(1) = 6.55, *p* = .011, but no further improvement as an interaction with condition. Faster processing speed sped up response times across all conditions (β = −0.01, *t* = −3.65, *p* = .011).

### Three-way interactions for idiom and cognitive variables

**Fig. 3:**
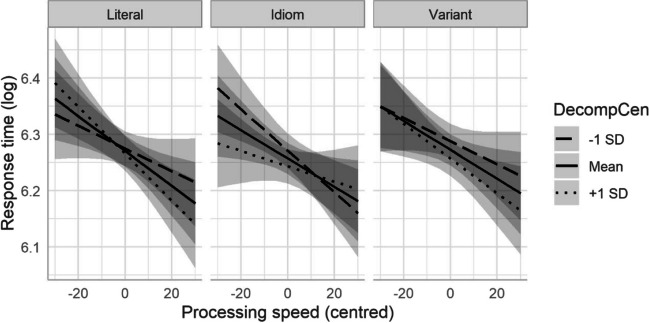
Three-way interactions between condition, decomposability, and processing speed, for log-transformed response times

We finally checked for any significant three-way interactions among the idiom and cognitive variables. The inclusion of a three-way interaction for condition, decomposability, and processing speed significantly improved the model, χ^2^(8) = 23.48, *p* = .003, compared with the inclusion of decomposability only. Figure 3 suggests that despite this interaction, differences between conditions were minor here.

## Discussion

We examined the way in which idiom variation is processed, focusing on the effects of properties of the idioms themselves and individual cognitive differences, and comparing two different methodologies that have been widely employed in the literature—eye tracking while reading and cross-modal priming.

### Eye tracking while reading

In the eye-tracking experiment, the widely reported idiom advantage was not observed in either early or later measures (idioms were not read any more quickly than literal controls).^6^ Of primary interest in this study was the comparison with variants: idioms modified to retain the same underlying metaphorical idea but presented in an unfamiliar lexical form. On first encounter, variants were read no differently to either idioms or literal control phrases, suggesting that readers were happy to interpret these at face value as literal statements. However, in later measures—reflecting overall time reading and rereading the critical phrase and time spent reading the rest of the sentence—a clear difference for variants emerged. The nature of the stimuli in the present study, where disambiguating context followed the target phrase, is critical here. Whilst readers saw nothing amiss with the variants on first encounter, once they had proceeded to a point where the meaning of the phrase needed to be integrated into the meaning of the whole sentence, additional processing for variants was required in order to make sense of what they had read. Two possibilities for this additional processing are (1) that the initial, literal parse of the variants was not sufficient to derive any sense from the sentence, hence a reanalysis was required; or (2) that readers were able to recognize and reconfigure the variants as modifications of known phrases, but the surprisal/novelty of these required additional processing time to resolve.

The first possibility is consistent with the studies discussed previously, where modification (especially lexical modification) generally causes difficulty and/or reduces the likelihood of idioms being recognized (Geeraert et al., 2017; Hamblin & Gibbs, 1999; Holsinger, 2013; McGlone et al., 1994; Smolka & Eulitz, 2020). Importantly, when studies have directly investigated online processing of idiomatic variation, evidence of slowed processing has tended to be most prominent in later measures (Haeuser et al., 2020; Kyriacou et al., 2020, 2021), as in the present results. Titone et al. (2019) made a distinction between earlier, “retrieval-based” and later “compositional” stages, which may further support the idea that the additional processing required for variants only emerged when more careful compositional analysis was required to make sense of the sentence as a whole. For variants, with nothing to “retrieve” on first encounter, participants had no option but to parse the phrase as literal, then reconsider this when the context demonstrated that this was problematic.

A second possibility is that readers had relatively little difficulty in recognizing variants and reconfiguring them, but the lower likelihood of the variant nouns following the verb may be reflected in the longer overall processing times. Clearly, idioms, as formulaic phrases, have higher transitional probabilities than variants (the likelihood, based on corpus frequency, that a given noun will follow a given verb; i.e. *play with* is much more likely to be followed by *fire* than *acid*), and various studies have demonstrated the effects of this variable in the eye-tracking record (Frisson et al., 2005; McDonald & Shillcock, 2003a, 2003b). Of note, Frisson et al. (2005) found effects of this in early measures in their data (as opposed to the exclusively later effects seen for our data), and also showed that transitional probability could not reliably be isolated from more general effects of predictability (e.g., from the sentence context). For these reasons, this explanation is perhaps less convincing for our results.

### Cross-modal priming

One aim of this study was to explicitly compare results from two widely used but rarely combined methodologies. Whilst eye tracking allows us to infer processing effects from reading patterns (but crucially, does not tell us directly how readers interpreted any given word or phrase), cross-modal priming offers a more direct measure of meaning activation. Given how widely this has been used to demonstrate the activation of idiom meanings in previous studies, it was surprising that we saw no “idiom superiority effect” in the present study. Since the magnitude of this effect does vary widely (from around 20 ms in Findlay & Carrol, 2019, to over 50 ms in Cacciari & Tabossi, 1988), it is possible that more studies may have found null results here, hence the near universality of this effect in the literature may reflect the so-called file drawer problem of unpublished null results (Rosenthal, 1979) and not necessarily give a true picture. We included the degree of relatedness between idioms and their target words in the analysis to control for any differences here, hence the lack of an effect was not simply due to the target words being too far removed from the intended figurative meanings of the idioms.

Importantly for this study, no difference at all was observed between literal and variant conditions, confirming at least that variant phrases generated no consideration of figurative meaning in the timeframe employed here (250 ms following the offset of the phrase). Blasko and Connine (1993) found (mixed) evidence that even low familiar metaphors showed some degree of priming for figuratively related targets at 750 ms post-offset, so it may be that the time course of our study was not sufficient to capture any effects for variants. Combined with the lack of early effects from the eye-tracking study, it is likely that participants simply treated the variants as literal phrases in the first instance. Any attempt to reconfigure/reinterpret these was time-consuming, as observed in the eye-tracking data, and clearly took longer than the 250 ms allotted in the cross-modal priming study.

### Effects of idiom and cognitive variables

Effects of other variables were limited in both the eye-tracking and cross-modal priming results.^7^ Surprisingly, given its overall importance in idiom processing, familiarity had no effects on any measures for any of the phrase types in either study. We hypothesized that this may have an effect in one of two ways: either more familiar idioms would be more entrenched and hence may be less amenable to variation (cf. Haeuser et al., 2020); alternatively, more familiar idioms may be better known and hence easier to reconfigure. Since neither pattern emerged, there is little more to conclude here, although the overall high levels of familiarity may mean that any potential for variation was masked here. Similarly, literal plausibility had no effect on any phrase type, but decomposability did demonstrate an effect in both studies. In the eye-tracking data, it affected post-phrase regression path durations (time spent reading the post-phrase region, plus any time spent rereading the phrase itself, before the trial ended), with more decomposable phrases from all conditions showing shorter regression paths. In the cross-modal priming study, decomposability sped up response times for all phrase types. In both cases, this suggests that when figurative and literal readings were more closely aligned (as in examples like *lift your spirits* / *lift your feelings*), processing was overall easier (for both idioms and variants) than when the figurative and literal readings were further apart (e.g., *walk on air* / *walk on wind*).

The cognitive variables explored here (working memory, processing speed and inhibitory control) likewise had limited effects on processing in the two studies. Given the somewhat mixed picture presented in the previous literature, this is not altogether surprising. Studies of metaphor processing have generally found an effect of variables such as working memory and inhibitory control, but idioms have sometimes shown these effects (e.g., Cacciari et al., 2018, for cross-modal priming) and sometimes not (e.g., Columbus et al., 2015, for eye-tracking). If variants were treated as novel metaphorical expressions, we would have expected them to show results more akin to those found for metaphors, but no such patterns were found for any of the eye-tracking measures observed here. The two variables that we most expected to have an effect based on previous studies were working memory (Blasko, 1999; Carriedo et al., 2016; Chiappe & Chiappe, 2007; Columbus et al., 2015; Kazmerski et al., 2003; Olkoniemi et al., 2016; Pierce et al., 2010) and inhibitory control (Cacciari et al., 2018; Papagno et al., 2003). Neither had an effect on idioms or variants in either study, which further suggests that variants were treated more like literal phrases on first encounter (i.e., no competition existed between figurative and literal meanings; hence, neither working memory nor inhibitory control came into play to help readers “commit” to one interpretation or the other). The one variable that did show an effect was processing speed, where faster processors read idioms and literal controls, but not variants, more quickly than slower processors (Fig. 2, right panel). As with the overall patterns, this effect was only apparent in later measures (total reading time). This may support the overall interpretation that variants were treated as literal on first encounter, but required significant later analysis (for all readers, regardless of natural processing speed) once the remaining sentence context had been encountered.

Similarly, in the cross-modal priming study, processing speed decreased response times across the board, but neither working memory nor inhibitory control made any improvement to the analysis. As argued above, this strengthens the assumption that variants were treated as literal phrases on first encounter, although effects of these variables may become apparent at a longer time course (see, e.g., the differential effects observed at different presentation speeds in Libben & Titone, 2014).

### Overall summary and conclusions

Existing literature on idiom variation shows a broad distinction between studies of interpretation, where idioms can still be successfully understood even when modified in various ways, and studies of online processing, where a clear cost is incurred by creative manipulation. In the present findings, that cost showed up in later measures of reading (generally thought to reflect meaning integration processes), but not in earlier measures. Of importance here is the fact that a comparison of early versus late measures, as we have adopted in this study, is a simplistic and possibly even misleading distinction (Clifton et al., 2007). For one, the measures we use are not independent of one another, hence a “late” measure like total reading time by definition also includes “early” measures such as first-pass reading time. This makes it much harder to neatly separate these into early (recognition) and later (meaning selection/integration) processes, and Vasishth et al. (2012) argue that critical effects may well be subject to a “lag,” whereby they start early but are only observable later in the processing record. Whilst it is therefore not possible to conclusively say that variants were initially treated as literal phrases, with subsequent processing only required at a later stage, it is clear that variants posed little difficulty for readers during the initial parse of the sentence but did induce subsequently more rereading than either literal controls or known idioms. In comparison, Carrol and Littlemore (2020) performed a similar study with entirely unfamiliar idioms and found significantly slower reading (compared with a literal paraphrase of the same meaning) in early as well as late measures (all at the *p* < .001 level, so these results would have been significant even with the more conservative analysis adopted in the present study). Tentatively, then, we conclude from the present study that the challenge posed by variants was one that primarily, although possibly not exclusively, manifested itself in the process of making sense of the phrase in context, rather than during the initial stages of lexical recognition and access.

With this in mind, we further assume that the additional total reading time observed for variants is a result of the need to reconsider the variants and assign an alternative figurative meaning, but may also reflect surprisal at an unfamiliar version of a known phrase. On balance, and for the reasons outlined earlier in this section, we favour the former interpretation of our results here. Importantly, there is little evidence that variants were treated as novel metaphors, given the lack of an effect of variables such as working memory, which have shown such consistent effects in the metaphor literature. In cross-modal priming, which directly tests the activation of meaning, no consideration of the figurative meaning of variants was observed, although the lack of an idiom advantage for canonical phrases means that this is unsurprising in the present study, and far from conclusive. The lack of clear and consistent effects for the cognitive variables studied here leaves the question of their contribution open, since previous studies have also found mixed results. The use of two complementary approaches here allowed us to directly compare methods, and both produced results that were less clear in terms of an idiom advantage than is generally reported in the literature.

### Supplementary Information

ESM 1 (DOCX 19 kb)

## Data Availability

All data and materials are available (https://osf.io/g54mb/).

## Appendix: Stimulus items used in both studies

**Table:**

Idiom	Variant	Literal control
*bite the dust*	*bite the dirt*	*bite the meat*
*blow your cover*	*blow your shelter*	*blow your nose*
*break the ice*	*break the shell*	*break the bowl*
*burn your bridges*	*burn your routes*	*burn your dinner*
*bury the hatchet*	*bury the cleaver*	*bury the embers*
*call the shots*	*call the moves*	*call the kids*
*change your tune*	*change your song*	*change your job*
*clear your name*	*clear your image*	*clear your books*
*cover your tracks*	*cover your steps*	*cover your plate*
*crack the whip*	*crack the belt*	*crack the pane*
*cut your losses*	*cut your debts*	*cut your grass*
*drag your feet*	*drag your legs*	*drag your bags*
*draw a blank*	*draw a zero*	*draw a clown*
*drop the ball*	*drop the baby*	*drop the glass*
*drown your sorrows*	*drown your sadness*	*drown your lilies*
*eat your words*	*eat your ideas*	*eat your food*
*face the music*	*face the songs*	*face the front*
*fall from grace*	*fall from esteem*	*fall from height*
*fan the flames*	*fan the blaze*	*fan the queen*
*get the picture*	*get the image*	*get the letter*
*grease the wheels*	*grease the chain*	*grease the bike*
*hit the road*	*hit the street*	*hit the door*
*hold the fort*	*hold the camp*	*hold the spoon*
*jump the gun*	*jump the shot*	*jump the fence*
*learn the ropes*	*learn the moves*	*learn the rules*
*lick your wounds*	*lick your injuries*	*lick your finger*
*lift your spirits*	*lift your feelings*	*lift your weights*
*lose the thread*	*lose the strand*	*lose the fight*
*meet your match*	*meet your equal*	*meet your uncle*
*miss the boat*	*miss the ship*	*miss the play*
*over the moon*	*over the stars*	*over the fence*
*pass the torch*	*pass the lamp*	*pass the dish*
*play with fire*	*play with acid*	*play with toys*
*pull some strings*	*pull some wires*	*pull some blankets*
*rock the boat*	*rock the ship*	*rock the chair*
*roll with the punches*	*roll with the blows*	*roll with the waves*
*save your skin*	*save your bones*	*save your cash*
*scratch the surface*	*scratch the shell*	*scratch the mirror*
*shake a leg*	*shake a foot*	*shake a tree*
*sing your praises*	*sing your merits*	*sing your tunes*
*sit on the fence*	*sit on the wall*	*sit on the grass*
*smell a rat*	*smell a toad*	*smell a rose*
*speak your mind*	*speak your views*	*speak your lines*
*spill the beans*	*spill the seeds*	*spill the juice*
*steal the show*	*steal the play*	*steal the gold*
*swallow your pride*	*swallow your dignity*	*swallow your tablet*
*throw in the towel*	*throw in the cloth*	*throw in the coins*
*turn the tables*	*turn the boards*	*turn the pages*
*under the weather*	*under the elements*	*under the bridge*
*walk on air*	*walk on wind*	*walk on stage*
*waste your breath*	*waste your oxygen*	*waste your petrol*

## Footnotes

1 In these studies, lexical variation took different forms, including replacement with a near synonym (*kick the bucket* → *kick the pail; kick the bucket* → *punt the bucket*), insertion of an additional element (*spill the beans* → *spill a single bean*), and blends of similar idioms (*go through the roof* + *off the charts* → *go through the charts*).

2 Blasko and Connine (1993) include “aptness” in their analysis, which is functionally equivalent to what others have called transparency or motivation, in that it asks how well a particular metaphor expresses its intended figurative meaning.

3 Although Haeuser et al. (2020) report the results of a range of cognitive tests to demonstrate differences between their participant groups, they did not directly use these scores as predictors in their main analysis.

4 Where this led to convergence issues, we removed random correlations between intercepts and slopes for items, then for subjects. All models converged after this.

5 Since the final noun is included in calculation of phrase total reading time, these measures are not independent.

6 Although note that previous studies employing eye tracking usually do not apply the more conservative approach to analysis employed here. With a less conservative approach, the difference in total reading time between idioms and literal phrases (*p* = .014) would be seen as significant.

7 We note here that the lack of clear and consistent idiom superiority effects, and the lack of effects for cognitive and idiom variable, may be a power issue, and future studies might usefully test much larger samples, especially when individual differences are being investigated.
