# Supplementary material for: As easy as cake or a piece of pie? Processing idiom variation and the contribution of individual cognitive differences
Source: Mem Cognit. 2023 Sep 19;52(2):334–51. doi: 10.3758/s13421-023-01463-x (PMC10896937; doi:10.3758/s13421-023-01463-x)
Supplement: Supplementary file 1 — (DOCX 19 kb) [file 13421_2023_1463_MOESM1_ESM.docx]

Supplementary materials

*Item development*

Idioms were selected based on three published sets of normed data: Titone and Connine (1994b), Libben and Titone (2008) and Bulkes and Tanner (2017). In total, these datasets comprise over 1000 phrases, although many of these appear on more than one list. To maintain a comparable structure, only items of the broad form “Verb-X-noun” were considered. In some cases the verb was replaced by a preposition (e.g. *over the moon*), and “X” was a definite or indefinite article (e.g. *kick the bucket*), possessive pronoun (e.g. *lose your mind*) or preposition (e.g. *play with fire*). In some cases more than one element appeared in the “X” position (e.g. *sit on the fence*), but all idioms matched the main criterion of having two main lexical items including a final noun. Since the lists were all compiled and normed in North America, we restricted selection to only those items that were also common in British English. These conditions led to an initial pool of 90 items. Decomposability and literal plausibility ratings were collected from the published studies, and where items appeared in more than one list, an average decomposability rating was obtained.^[[1]](#footnote-1)^

We created a plausible variant for each phrase by replacing the final noun in a way that maintained the metaphorical sense of the idiom. For example, in *play with fire*, we replaced *fire* with *acid*, to maintain the underlying meaning of *play with* [*dangerous thing*]. For each item, we calculated a measure of semantic distance between idiom and variant nouns using the ADW tool (a WordNet based approach to measuring semantic similarity available at: [lcl.uniroma1.it/adw/](http://lcl.uniroma1.it/adw/)). A literal control phrase for each idiom was also created in the same way, hence for *play with fire* the control was *play with toys*. Nouns were matched as closely as possible for length and individual frequency (based on the British National Corpus), and items where this was not possible (where a plausible matched noun could not be found for both the variant and control phrases) were discarded.

We placed the remaining 70 items into a norming study to assess acceptability for the variants. Participants (n = 17, 14 females, mean age = 18.7, SD = 0.6, all native speakers of British English) were presented with each phrase and its variant and told the figurative meaning, then asked to rate on a 5-point scale how much the variant could be considered to convey the same meaning as the original. We eliminated any extremely non-decomposable idioms at this point (e.g. *kick the bucket*, *cut the mustard*), as these obtained very low substitutability scores.

A final list contained a total of 51 items, selected to give a range of scores on the variables identified above. These varied in terms of their decomposability rating (out of 5: mean = 2.54, SD = 1.12, range = 0.59-4.74), substitutability rating (out of 5: mean = 3.06, SD = 0.65, range = 1.9-4.5) and semantic similarity (ADW) scores (out of 1.00: mean = 0.54, SD = 0.27, range = 0.13-1.00). Substitutability was significantly correlated with both decomposability (*r*(49) = .43, *p* = .002) and semantic similarity (*r*(49) = .34, *p* = .014), meaning that variants of more decomposable idioms, and those where the final noun for an idiom and its variant were semantically closer, were judged to be closer to the idiomatic meaning. Repeated measures ANOVAs for the final lists showed no differences among idiom, variant and literal nouns for length (*f*(2) = 0.599, p = .551) or frequency (measured on the Zipf scale; *f*(2) = 0.096, *p* = .909).

These items were placed into a second norming study to collect decomposability ratings, firstly for the purposes of comparing with those taken from the published norms, and secondly to compare judgments for idioms vs. variants. The items were presented in two counterbalanced lists so that participants (n = 28, 25 females, mean age = 18.7, SD = 0.76) saw either an idiom or its variant, along with the figurative meaning. They were told that some phrases would be less common than others (to account for variants being unfamiliar) and asked to judge on a 5-point scale how much the component words contributed to the overall meaning presented. The ratings for idioms (mean = 3.06, SD = 0.77) and variants (mean = 2.73, SD = 0.78) were significantly correlated (*r* = 0.663, *p* < .001), although a paired samples t-test showed that the idioms were rated as consistently more decomposable than the variants (*t*(50) = 3.709, *p* < .001). This likely reflects an effect of familiarity, whereby speakers over-estimate the decomposability of idioms they know well (e.g. Carrol et al., 2018; Keysar & Bly, 1995). We also compared idiom ratings obtained here with the average decomposability ratings taken from the published norming data, and found a significant correlation (*r* = 0.728, *p* < .001), suggesting that these ratings are relatively stable.

For the reading study, context sentences were created to support either the idiom or its variant (hence sentences were always the same for idioms/variants), or to support the literal version of the phrase. All sentences consisted of two conjoined clauses. The first clause always began with a personal pronoun and introduced the target phrase in 4-6 words in a neutral way, followed by the target phrase, e.g. *They have both been playing with … fire / acid / toys*. The second clause followed a conjunction (*and* or *but*) and was consistent with the figurative (e.g. *and it's no surprise that things have gone badly*) or literal (e.g. *and seem to have been getting on very well together*) version of the phrase. Sentences were therefore identical in terms of the pre-target region (length in characters, mean = 18.8, SD = 2.2), and post-target regions were of comparable lengths (idiom / variant sentences: mean = 48.8, SD = 2.4; literal sentences: mean = 49.0, SD = 2.4; *t*(50) = 1.22, *p* = .228). A set of 51 filler items was created, comprising literal sentences of approximately the same length and syntactic structure (two conjoined clauses) as the critical sentences. Items were counterbalanced over three presentation lists, with an idiom, its variant and its literal control phrase appearing on different lists. Each list contained 17 items in each condition, and the same filler sentences were used in all three presentation lists, to give a total of 102 items per list.

For the cross-modal priming study, a word related to the figurative meaning of the idiom was chosen (e.g. for *play with fire* the target was ‘danger’), matched as far as possible across all items for length (mean = 6.00 characters, SD = 0.96, range = 4-8) and frequency (measured on the Zipf scale: mean = 4.54, SD = 0.38, range = 3.70-5.37). To confirm that these were sufficiently related to the figurative meaning, an online norming study was undertaken where a group of English native speakers (n = 30, 23 females, mean age = 21.7, SD = 2.4) were presented with each phrase and the target word, then asked to rate on a 5-point scale how closely related the word was to the meaning of the whole phrase. Average relatedness rating was 3.9 (SD = 0.4), with all items rated above 2.9/5.

The sentences created for the reading task were all used for the cross-modal priming task, up to the end of the key phrase, i.e. for *play with fire* the stimulus item in the idiom condition was *They have both been playing with fire*. Mean length of the items was 7.5 words (SD = 0.6). A set of filler items based on the fillers from the reading task was also created by cutting the sentence off at after the first clause, to leave items of around the same length as the critical stimuli (mean = 6.1 words, SD = 1.1). We also added in an additional 17 idioms (unused items from the item selection procedure, placed into comparable short sentence fragments) to act as fillers, to give a total of 68 fillers. The purpose of this was to ensure that some (non-critical) idioms could be followed by a pseudoword, to avoid participants from developing expectancy-based strategies during the task. The critical and filler items were then used to create sound files using an online voice synthesiser (www.fromtexttospeech.com), which would ensure that any unconscious cues as to the meaning of any of the stimuli were avoided (see e.g. Van Lancker, Canter & Terbeek, 1981). All stimuli were recorded with a female voice with a British English accent, and sound files were checked for accuracy of pronunciation to ensure that all words appeared as they should. Files were converted from mp3 to WAV using an online audio convertor (online-audio-converter.com).

Critical items were paired with their related word and counterbalanced over the same three presentation lists as in the reading study. Filler items were always paired with a pseudoword, meaning that overall 17 idioms (critical items), 17 variants and 17 literal controls were followed by a real (related) word, and 68 fillers (51 literal sentences and 17 idioms) were followed by a pseudoword. Pseudowords were all pseudohomophones between 4 and 8 letters long (mean = 5.3, SD = 0.9), created using the ARC Nonword Database (Rastle, Harrington & Coltheart, 2002).

Participants were randomly assigned to one of the combinations of presentation lists (AB, AC, BC) so that no-one saw the same list for the two main tasks.

1. The three norming papers operationalised and rated decomposability in slightly different ways, so we normalised this prior to calculating an aggregate. [↑](#footnote-ref-1)
